# Scalable true random number generator using adiabatic superconductor logic

**DOI:** 10.1038/s41598-022-24230-5

**Published:** 2022-11-21

**Authors:** Wenhui Luo, Olivia Chen, Nobuyuki Yoshikawa, Naoki Takeuchi

**Affiliations:** 1 Department of Electrical and Computer Engineering, Yokohama National University, Yokohama, Kanagawa 240-8501 Japan; 2 Department of Computer Science, Tokyo City University, Setagaya, Tokyo, 158-8557 Japan; 3 Institute of Advanced Sciences, Yokohama National University, Yokohama, Kanagawa 240-8501 Japan; 4 Research Center for Emerging Computing Technologies, National Institute of Advanced Industrial Science and Technology (AIST), Tsukuba, Ibaraki 305-8560 Japan

**Keywords:** Electrical and electronic engineering, Computer science, Applied physics

## Abstract

Alternative computing such as stochastic computing and bio-inspired computing holds promise for overcoming the limitations of von Neumann computers. However, one difficulty in the implementation of such alternative computing is the need for a large number of random bits at the same time. To address this issue, we propose a scalable true-random-number generating scheme that we refer to as XORing shift registers (XSR). XSR generates multiple uncorrelated true random bitstreams using only two true random number generators as entropy sources and can thus be implemented by a variety of logic devices. Toward superconducting alternative computing, we implement XSR using an energy-efficient superconductor logic family, adiabatic quantum-flux-parametron (AQFP) logic. Furthermore, to demonstrate its performance, we design and observe an AQFP-based XSR circuit that generates four random bitstreams in parallel. The results of the experiment confirm that the bitstreams generated by the XSR circuit exhibit no autocorrelation and that there is no correlation between the bitstreams.

## Introduction

General-purpose von Neumann computers are approaching their performance limit due to the end of Moore’s law and Dennard scaling^1^. As a consequence, alternative computing technologies are being extensively investigated. Of note is the fact that many of the alternative computing technologies (e.g., stochastic computing^2–4^, simulated annealing^5–7^, bio-inspired computing^8–10^, and invertible logic^11–13^) exploit stochastic operations to gain computing performance and require many random bits at the same time. Hence, in order to build computing systems based on alternative computing, the question of how to generate and distribute multiple random bitstreams in parallel in the entire system is of significant concern. In the case of typical semiconductor circuitry, a random bitstream is generated by a pseudo-random number generator (PRNG) such as a linear-feedback shift register^14,15^, and a large number of PRNGs are used to generate multiple random bitstreams in parallel, resulting in high energy and hardware overhead. For example, in IBM’s neuromorphic system TrueNorth^9^, 27% of the logic gates in each neuron circuit are used for a PRNG. Furthermore, since a bitstream generated by a PRNG has a finite length, the seed (i.e., initial state) of each PRNG needs to be carefully selected to avoid correlation between bitstreams^16^, which can adversely affect the accuracy of computation. Therefore, to implement alternative computing, logic devices that can generate uncorrelated true random bitstreams in an energy- and hardware-efficient manner are required.

In recent years, we have been developing adiabatic quantum-flux-parametron (AQFP) logic^17,18^. The AQFP is an energy-efficient logic device based on the quantum flux parametron^19,20^ that can operate with extremely small energy dissipation near the thermodynamic limit^21^ due to an energy-efficient switching scheme, adiabatic switching^22–24^. Moreover, the AQFP can easily perform stochastic operations by thermal fluctuations^25,26^. As part of our effort, we have developed a true random number generator (TRNG) using AQFP logic and demonstrated the generation of a low-autocorrelation random bitstream^27^. Thus, AQFP logic appears highly suitable as a building block for implementing alternative computing. The next step towards large-scale alternative computing-based systems is to develop a scheme to generate multiple random bitstreams in a scalable way using AQFP logic.

In the present study, we propose a scalable true-random-number generating scheme that we refer to as XORing shift registers (XSR) and implement XSR using AQFP logic. XSR generates multiple uncorrelated true random bitstreams in parallel using only two TRNGs as entropy sources. This is a huge advantage in the development of large-scale systems. In general, a TRNG is a somewhat complex circuit (various TRNGs can be found in the literature^28–33^), and minimizing their number is highly desirable. Since XSR utilizes XOR gates to generate multiple random bitstreams, we first explain random number generation using XOR gates. We then explain the operating principle of XSR and how to implement XSR using AQFP logic. Finally, we experimentally demonstrate an AQFP-based XSR circuit that generates four random bitstreams, each of which has no autocorrelation or correlation with the other bitstreams. Our results indicate the path towards scalable, energy-efficient alternative computing-based systems using AQFP logic.

## Random number generation using XOR gates

Table 1 describes the truth table of an XOR gate, where *A* and *B* are the inputs and *X* (= *A* ⊕ *B*) is the output. Hereafter *A* and *B* are assumed to be uncorrelated random bits (a random bit becomes a 0 or 1 stochastically with the same probability).

**Table 1:** Truth table of an XOR gate.

*A*	*B*	*X* = *A* $\oplus$ *B*
0	0	0
0	1	1
1	0	1
1	1	0

### Logical viewpoint

Most importantly, XORing two random bits generates another random bit^34^ as follows: *A* and *B* are random bits, so that the four possible input combinations [(*A*, *B*) ∈ {(0, 0), (0, 1), (1, 0), (1, 1)}] appear randomly. Consequently, *X* becomes 0 or 1 randomly (i.e., *X* is also a random bit), since *X* includes the same number of 0 s as the number of 1 s in the truth table. Here we discuss the correlation regarding *A*, *B*, and *X* by calculating mutual information^35^, which quantifies the correlation between probability variables. The mutual information between *A* and *X* is given by

1 $$I\left(A;X\right)=H\left(A\right)+H\left(X\right)-H\left(A,X\right)$$

where *H*(*A*) and *H*(*X*) are the logical entropy (i.e., Shannon entropy of the logic states)^35^ of *A* and *X*, respectively, and *H*(*A*, *X*) is the joint logical entropy of *A* and *X*. *H*(*A*) is given by

2 $$H\left(A\right)=-\sum_{a}P\left(a\right){\text{ln}}P\left(a\right)$$

where *A* takes a value *a* with the probability *P*(*a*). According to Table 1, *a* ∈ {0, 1} and *P*(0) = *P*(1) = 0.5, which gives *H*(*A*) = ln2. Similarly, *H*(*X*) =  − Σ_*x*_*P*(*x*)ln*P*(*x*) = ln2, and *H*(*A*, *X*) =  − Σ_*a*,*x*_*P*(*a*, *x*)ln*P*(*a*, *x*) = 2ln2. As a result, *I*(*A*; *X*) = ln2 + ln2 − 2ln2 = 0, which indicates that *A* and *X* are not correlated with each other, i.e., one cannot tell the value of *A* from a given value of *X*, and vice versa. Likewise, *I*(*B*; *X*) = 0 and *I*(*A*; *B*) = 0. Therefore, there is no correlation between any pair of *A*, *B*, and *X*. However, *A*, *B*, and *X* are correlated since, if one knows the values of any two of the three (*A*, *B*, and *X*), one can tell the value of the other. This is quantified by the mutual information among *A*, *B*, and *X* as follows:

3 $$I\left(A;B;X\right)=H\left(A\right)+H\left(B\right)+H\left(X\right)-H\left(A,B\right)-H\left(A,X\right)-H\left(B,X\right)+H\left(A, B,X\right)$$

*H*(*A*) = *H*(*B*) = *H*(*X*) = ln2, and *H*(*A*, *B*) = *H*(*A*, *X*) = *H*(*B*, *X*) = *H*(*A*, *B*, *X*) = 2ln2. Consequently, *I*(*A*; B; *X*) =  − ln2. The above discussion indicates that an XOR gate can increase two uncorrelated random bits (*A* and *B*) to three uncorrelated random bits (*A*, *B*, and *X*), where correlation appears only when all of *A*, *B*, and *X* are taken into account together.

### Thermodynamic viewpoint

In physical systems, random number generation is related to thermodynamics because logical entropy is tied to (thermodynamic) entropy: in the quasi-static limit, Δ*H* = Δ*S* = β*Q*^35,36^, where Δ*H* is the logical entropy change of the system, Δ*S* is the entropy change of the system, β is inverse temperature, and *Q* is the heat absorbed by the system. For instance, an AQFP TRNG^27^ generates a random bit (i.e., Δ*H* = ln2) by increasing entropy via heat absorption (i.e., Δ*S* = β*Q* = ln2)^25^. Thus, we explore random number generation using XOR gates from the thermodynamic viewpoint.

We first derive the thermodynamic relations for a logic gate with two uncorrelated random inputs (*A* and *B*) and an output (*X*). From Eq. (3), the total logical entropy change during a logic operation is given by

4 $$\Delta H\left(A, B,X\right)=\Delta H\left(A,B\right)+\Delta H\left(A,X\right)+\Delta H\left(B,X\right)-\Delta H\left(A\right)-\Delta H\left(B\right)-\Delta H\left(X\right)+\Delta I\left(A;B;X\right)$$

The inputs do not change during a logic operation, so that Δ*H*(*A*) = Δ*H*(*B*) = Δ*H*(*A*, *B*) = 0. Moreover, the total logical entropy change is linked to heat absorption. Hence, in the quasi-static limit (i.e., assuming that the logic operation is performed without energy dissipation), Eq. (4) becomes

5 $$\Delta H\left(A, B,X\right)=\Delta {H}_{\text{eff}}\left(X\right)+\Delta I\left(A;B;X\right)=\beta Q$$

where Δ*H*_eff_(*X*) = Δ*H*(*A*, *X*) + Δ*H*(*B*, *X*) − Δ*H*(*X*) is the effective logical entropy change of *X*; Δ*H*_eff_(*X*) becomes ln2 when *X* is a random bit that is not correlated with *A* or *B*. Conventional logic gates operate deterministically and do not include entropy-increasing processes such as heat absorption; thus, *Q* = 0 and Eq. (5) is reduced to

6 $$\Delta {H}_{\text{eff}}\left(X\right)=-\Delta I\left(A;B;X\right)$$

This equation shows that even if a logic gate does not include entropy-increasing processes, the logic gate can generate a random bit by producing mutual information.

**Figure 1:**
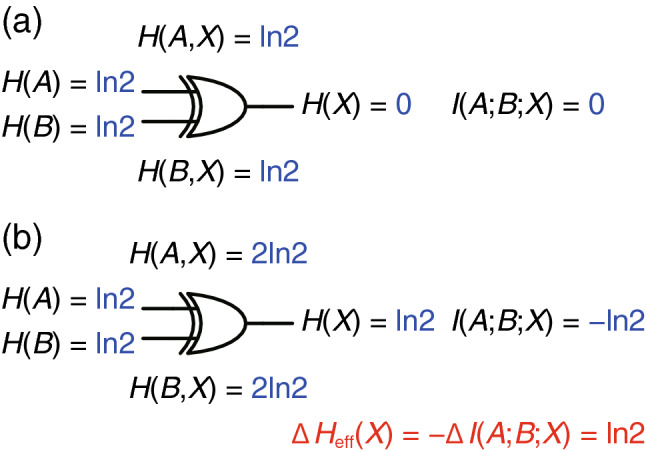
Logical entropy and mutual information regarding an XOR gate. (**a**) Initial state, where the output *X* is not generated. (**b**) Final state, where *X* (a random bit) is generated by producing mutual information *I*(*A*; *B*; *X*).

Figure 1 shows the change in logical entropy and mutual information regarding an XOR gate. *A* and *B* are random bits, so that *H*(*A*) = *H*(*B*) = ln2. In the initial state (Fig. 1a), *X* is not yet generated and thus *H*(*X*) = 0, which results in *H*(*A*, *X*) = *H*(*B*, *X*) = ln2 and *I*(*A*; *B*; *X*) = 0. In the final state (Fig. 1b), *X* is calculated from *A* and *B*: *H*(*X*) = ln2. As mentioned above, any pair of *A*, *B*, and *X* are not correlated with each other, but *A*, *B*, and *X* are correlated; thus, *H*(*A*, *X*) = *H*(*B*, *X*) = 2ln2 and *I*(*A*; *B*; *X*) =  − ln2. Consequently, Δ*H*_eff_(*X*) =  − Δ*I*(*A*; *B*; *X*) = ln2, which indicates that the XOR gate generates a random bit by producing mutual information and that XOR gate-based random number generation agrees with thermodynamics.

## XORing shift registers (XSR)

**Figure 2:**
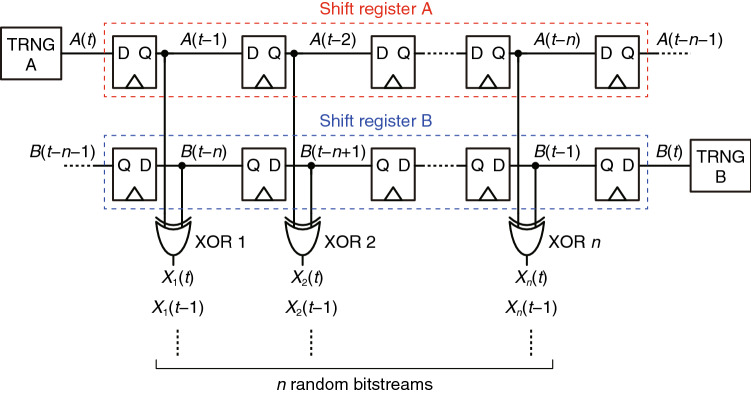
XSR. The two shift registers and *n* XOR gates generate *n* uncorrelated random bitstreams from the two uncorrelated random bitstreams generated by the two TRNGs.

XSR generates multiple random bitstreams in parallel based on random number generation using XOR gates. Figure 2 shows XSR generating *n* random bitstreams (*n* ∈ ℕ). The clock lines to the flip-flops are omitted for simplicity, and *t* is time in clock cycles. As indicated, XSR involves only simple circuits: two uncorrelated TRNGs (TRNGs A and B), two (*n* + 1)-bit shift registers (shift registers A and B), and *n* XOR gates (XOR 1, XOR 2, ..., XOR *n*). Shift register A transmits the random bitstream from TRNG A [*A*(*t*), *A*(*t* − 1), . . ., *A*(*t* − *n* − 1)], whereas shift register B transmits the random bitstream from TRNG B [*B*(*t*), *B*(*t* − 1), ..., *B*(*t* − *n* − 1)]. The *n* XOR gates produce *n* random bits [*X*_1_(*t*), *X*_2_(*t*), ..., *X*_*n*_(*t*)] in parallel, and each XOR gate generates a random bitstream; for instance, XOR 1 produces a random bitstream of *X*_1_(*t*), *X*_1_(*t* − 1), *X*_1_(*t* − 2), and so forth.

The outputs of the XOR gates can be described as follows: First, the output *X*_*i*_(*t*) (*i* ∈ {1, 2, ..., *n*}) from each XOR gate is a random bit since, as mentioned above, XORing two random bits produces another random bit; for instance, *X*_1_(*t*) is produced by XORing two random bits *A*(*t* − 1) and *B*(*t* − *n*) and is thus a random bit. Furthermore, the output bitstream from each XOR gate [*X*_*i*_(*t*), *X*_*i*_(*t* − 1), *X*_*i*_(*t* − 2),...] does not exhibit autocorrelation because each output in the bitstream is generated from different random bit pairs; for instance, *X*_1_(*t*) is generated from *A*(*t* − 1) and *B*(*t* − *n*) whereas *X*_1_(*t* − 1) is generated from *A*(*t* − 2) and *B*(*t* − *n* − 1), so that *X*_1_(*t*) is not correlated with *X*_1_(*t* − 1). Moreover, there is no correlation between the output bitstreams from different XOR gates. The important thing is that none of the outputs from the XOR gates are generated from the same random bit pairs, and that the output of an XOR gate is correlated with the inputs only when both inputs are taken into account; thus, correlation does not appear even if some outputs share the same random bit as an input. For instance, *X*_1_(*t*) is generated from *A*(*t* − 1) and *B*(*t* − *n*), and *X*_2_(*t* − 1) is generated from *A*(*t* − 3) and *B*(*t* − *n*); i.e., *X*_1_(*t*) and *X*_2_(*t* − 1) share *B*(*t* − *n*) as an input. However, *X*_1_(*t*) is not correlated with *X*_2_(*t* − 1) because *B*(*t* − *n*) is not correlated with *X*_1_(1) or *X*_2_(*t* − 1).

The above discussion establishes that each XOR gate in XSR generates a random bitstream without autocorrelation, and that there is no correlation between the random bitstreams from different XOR gates. Thus, XSR can generate many uncorrelated true random bitstreams in parallel using only two TRNGs. Moreover, since XSR utilizes simple logic gates, it can be easily implemented by various logic devices, including conventional semiconductor devices and emerging devices such as superconductor logic families. This is a significant advantage over the previously reported scheme^37^, which distributes random bits using asynchronous data collision with a careful timing design.

Note that XSR can be implemented using only one TRNG; for instance, XSR operates when TRNG B is removed and shift registers A and B are connected to each other. However, in this case, the autocorrelation of the TRNG may affect the quality of the generated random bit streams. Moreover, in general it is difficult to completely remove the autocorrelation of a TRNG^27^. Therefore, we decided to use two TRNGs in the present study.

## XSR using AQFP logic

**Figure 3:**
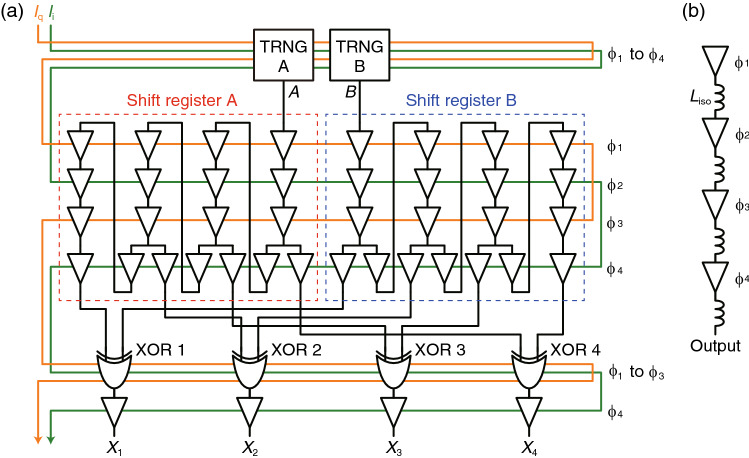
(**a**) AQFP-based XSR circuit for *n* = 4. The entire circuit is powered and clocked by the paired excitation currents, *I*_q_ and *I*_i_. (**b**) AQFP TRNG. The first buffer generates random bits because no input signal is applied, and the following buffers transmit the random bits to other circuits.

WE implement XSR using AQFP logic. AQFP logic gates are powered and clocked by ac excitation currents, so that special clocking schemes^38,39^ are needed to operate AQFP circuits. In the present study, we use the most common clocking scheme, four-phase clocking^38^, to operate the AQFP circuits. Figure 3a shows an example of an AQFP-based XSR circuit for *n* = 4. The entire circuit is clocked by the paired excitation currents, *I*_q_ and *I*_i_, with a phase separation of 90°. Logic operations are performed along the excitation phases, ϕ_1_ through ϕ_4_, with a phase separation of 90°. Consequently, the circuit shown in Fig. 3a operates in the same manner as that shown in Fig. 2, i.e., each XOR gate generates an uncorrelated random bit *X*_*i*_ (*i* ∈ {1, 2, 3, 4}) at every clock (excitation) cycle.

The circuit blocks can be explained as follows: A shift register is a buffer chain with feedback lines from ϕ_4_ to ϕ_1_, which enable data to transmit through a shift register in synchronization with the excitation phases. As shown in Fig. 3b, a TRNG^27^ is a buffer chain without an input. The first buffer generates a random bit at every clock cycle because no input signal is applied and the logic state is determined by thermal fluctuations. The following buffers transmit the random bits from the first buffer to other circuits. An isolation inductor *L*_iso_ is placed between each adjacent pair of buffers to mitigate the back action from the following circuits to the first buffer. An XOR gate^40^ comprises two splitters, two AND gates, and an OR gate, and thus requires three excitation phases.

**Figure 4:**
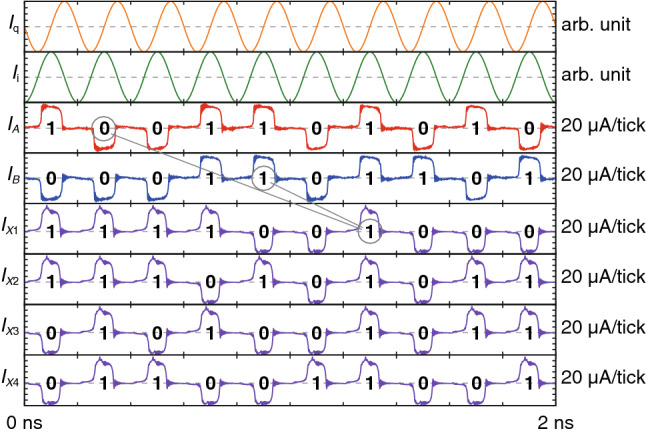
Simulation waveforms of the AQFP-based XSR circuit for *f* = 5 GHz. Four random bitstreams (*I*_*X*1_ through *I*_*X*4_) are generated from two random bitstreams (*I*_*A*_ and *I*_*B*_).

We conducted numeral simulations of the AQFP-based XSR circuit shown in Fig. 3a using a Josephson circuit simulator, JSIM_n^41–43^, with the device parameters based on the AIST 10 kA/cm^2^ Nb high-speed standard process (HSTP)^38^. Figure 4 shows the simulation waveforms of the AQFP-based XSR circuit for a clock frequency *f* of 5 GHz, where *I*_*A*_ and *I*_*B*_ are the signal currents representing the outputs of TRNGs A and B (*A* and *B* in Fig. 3a), respectively, and *I*_*X*1_ through *I*_*X*4_ are the signal currents representing the outputs of XOR 1 through XOR 4 (*X*_1_ through *X*_4_ in Fig. 3a), respectively. This figure clearly shows that four random bitstreams are generated from two TRNGs in synchronization with *I*_q_ and *I*_i_. Here we take a look at one of the outputs to see if the outputs are generated as expected. The circled 1 in *I*_*X*1_ is generated by XORing the circled 0 in *I*_*A*_ and the circled 1 in *I*_*B*_; *I*_*X*1_ lags behind *I*_*A*_ and *I*_*B*_ by five and two clock cycles (twenty and eight phases), respectively, which agrees with the excitation phases shown in Fig. 3a. The autocorrelation of each output bitstream and the correlation between the output bitstreams will be experimentally evaluated in the next section.

Here we estimate the Josephson junction count and power dissipation of the AQFP-based XSR as functions of *n*. As shown in Fig. 3a, a shift register includes five buffers per bit, except for the last bit slice including four buffers. Thus, shift registers A and B include approximately 10*n* buffers, which results in 20*n* Josephson junctions. An XOR gate includes 22 junctions^40^, so that the XOR gate array includes 22*n* junctions. Therefore, the XSR circuit includes approximately 42*n* Josephson junctions, where the junctions in TRNGs A and B and those in the buffers in the last excitation stage are ignored. Based on the previous study^21^, the energy dissipation of an AQFP circuit is roughly given by 1.4 × 10^−21^ J per junction at 5 GHz operation. Hence, the power dissipation of the XSR circuit is approximately 290*n* (pW) at 5 GHz operation.

## Experiments

**Figure 5:**
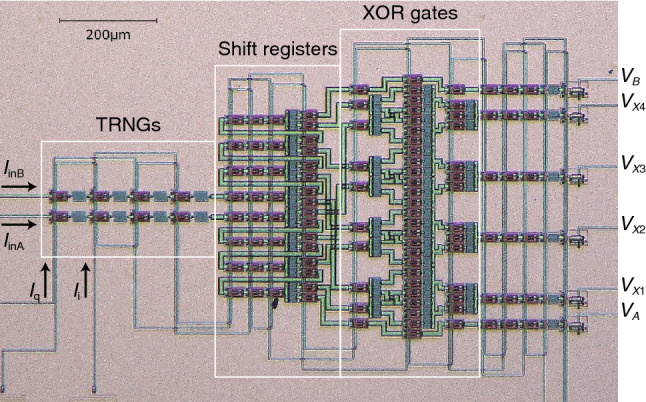
Micrograph of the AQFP-based XSR circuit for *n* = 4, which corresponds to the circuit shown in Fig. 3a. The circuit was fabricated using the HSTP.

AS a proof of concept, we fabricated an AQFP-based XSR circuit for *n* = 4 and observed its performance. Figure 5 shows a micrograph of the XSR circuit fabricated with the HSTP, based on the schematic diagram in Fig. 3a. The paired excitation currents (*I*_q_ and *I*_i_) were externally provided by a function generator. The outputs of the circuit (*X*_1_ through *X*_4_ in Fig. 3a) were converted into the corresponding voltage signals (*V*_*X*1_ through *V*_*X*4_) by dc superconducting interference quantum devices for read-out. The outputs of TRNGs A and B (*A* and *B* in Fig. 3a) were bypassed and also converted into voltage signals (*V*_*A*_ and *V*_*B*_). Input currents *I*_inA_ and *I*_inB_ were used to adjust the probability distribution of TRNGs A and B, respectively. Ideally, an AQFP TRNG generates 0 s and 1 s with the same probability. However, the probability distribution can vary due to circuit parameter variation; thus, *I*_inA_ and *I*_inB_ were applied so that 0 s and 1 s would appear with the same probability^27^. This suggests the importance of XSR; if *n* random bitstreams are generated from *n* AQFP TRNGs without using XSR, *n* input lines are needed to adjust the probability distribution of the TRNGs, which can deteriorate the scalability of the entire system.

**Figure 6:**
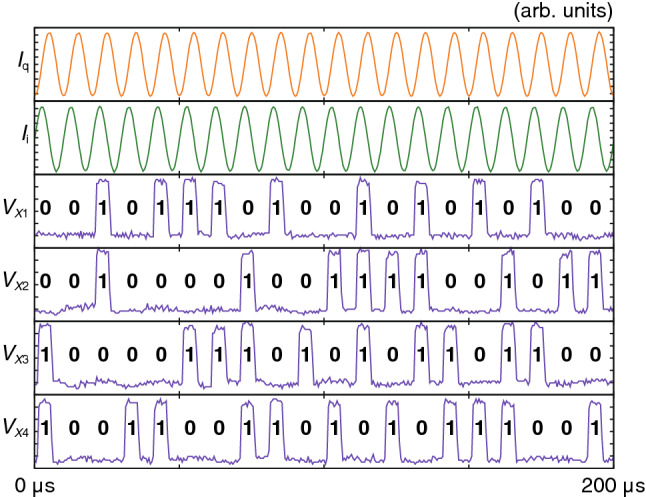
Measurement waveforms of the AQFP-based XSR circuit for *f* = 100 kHz. The outputs are converted into the corresponding voltage signals (*V*_*X*1_ through *V*_*X*4_).

We implemented the XSR circuit chip in the dipping probe and tested the chip in liquid He at 4.2 K and a low clock frequency (*f* = 100 kHz) due to the narrow bandwidth of the probe. First, we observed *V*_*A*_ and *V*_*B*_ and set *I*_inA_ and *I*_inB_ to 8.4 μA and 2.8 μA, respectively, so that 0 s and 1 s appear with the same probability. We then observed *V*_*X*1_ through *V*_*X*4_ to evaluate correlation. Figure 6 provides an example of the measurement waveforms of the XSR circuit, which clearly shows that four random bitstreams (*V*_*X*1_ through *V*_*X*4_) are generated in synchronization with *I*_q_ and *I*_i_. The measured operating margins of *I*_q_ and *I*_i_ were ± 25% and ± 24%, respectively.

### Autocorrelation of each bitstream

**Figure 7:**
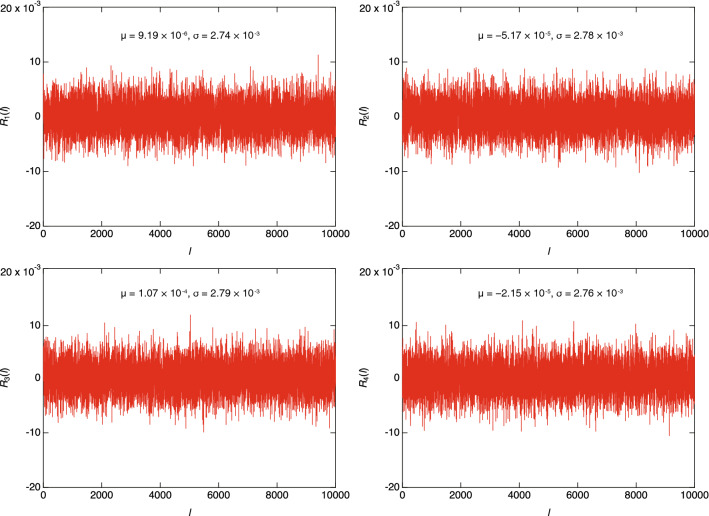
Measured autocorrelation functions of the bitstreams for *N* = 2^17^. For each autocorrelation function, the average is close to zero and the standard deviation is approximately 2.7 × 10^−3^ or 2.8 × 10^−3^, which indicates that each bitstream generated by XSR does not exhibit autocorrelation.

To evaluate autocorrelation, we measured the autocorrelation function *R*_*i*_(*l*) of each bitstream (*X*_*i*_) using the following equation:

7 $${R}_{i}\left(l\right)=\frac{1}{N}\sum_{t=0}^{N-1}{Y}_{i}\left(t\right)\cdot {Y}_{i}\left(t+l\right)$$

where *i* ∈ {1, 2, 3, 4}, *N* is the number of bits in the bitstream, *Y*_*i*_(*t*) is the normalized *X*_*i*_ [*Y*_*i*_(*t*) =  − 1 represents a logic 0 and *Y*_*i*_(*t*) = 1 represents a logic 1] at time *t*, in clock cycles, and *l* ∈ ℕ is the time lag. Figure 7 shows the measured *R*_*i*_(*l*) for *N* = 2^17^ and *l* ranging from 1 to 10^4^. All the |*R*_*i*_(*l*)| values are much smaller than 1 for the entire range of *l*. Furthermore, for each *R*_*i*_(*l*), the average μ is close to zero and the standard deviation σ is approximately 2.7 × 10^−3^ or 2.8 × 10^−3^. This confirms that each bitstream exhibits no autocorrelation since, if *X*_*i*_ is without autocorrelation, *Y*_*i*_(*t*)·*Y*_*i*_(*t* + l) becomes − 1 or 1 randomly and results in μ = 0 and σ = (*N*)^−0.5^  = 2.76 × 10^−3^ for *N* = 2^17^, which agrees with our measurement results. In sum, the above results show that an AQFP-based XSR circuit can generate multiple random bitstreams without autocorrelation from two TRNGs.

### Correlation between bitstreams

**Figure 8:**
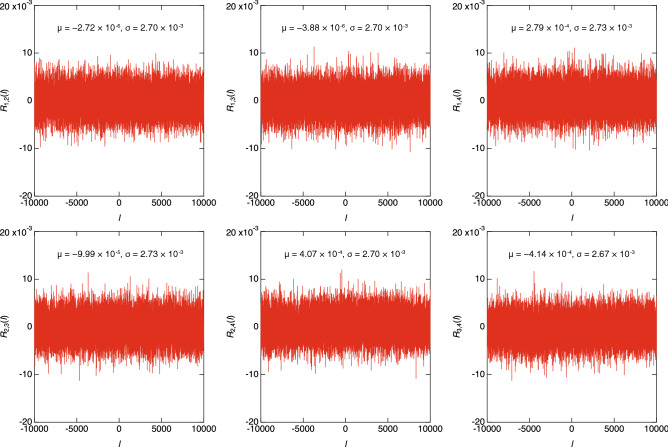
Measured cross-correlation functions between the bitstreams for *N* = 2^17^. For each cross-correlation function, the average is close to zero and the standard deviation is approximately 2.7 × 10^−3^, which indicates that there is no correlation between the bitstreams generated by XSR.

TO evaluate the correlation between the bitstreams generated by the XSR circuit, we measured the cross-correlation function *R*_*i*,*j*_(*l*) for each pair of the bitstreams (*X*_*i*_ and *X*_*j*_) using the following equation:

8 $${R}_{i,j}\left(l\right)=\frac{1}{N}\sum_{t=0}^{N-1}{Y}_{i}\left(t\right)\cdot {Y}_{j}\left(t+l\right)$$

where *i* ∈ {1, 2, 3, 4}, *j* ∈ {1, 2, 3, 4}, and *i* ≠ *j*. Figure 8 shows the measured *R*_*i*,*j*_(*l*) for *N* = 2^17^ and *l* ranging from − 10^4^ to 10^4^. All the |*R*_*i*,*j*_(*l*)| values are much smaller than 1 for the entire range of *l*. Furthermore, μ is close to zero and σ is approximately 2.7 × 10^−3^ for each *R*_*i*,*j*_(*l*), which demonstrates that there is no correlation between the bitstreams for the same reason as that given in the preceding discussion of autocorrelation (i.e., μ = 0 and σ = 2.76 × 10^−3^ if there is no correlation between the bitstreams). Taken together, these results show that an AQFP-based XSR circuit can generate multiple uncorrelated random bitstreams from two TRNGs.

## Conclusions

WE proposed a scalable true-random-number generating scheme, XSR, suitable for alternative computing. XSR generates multiple true random bitstreams in parallel using only two TRNGs, two shift registers, and a number of XOR gates. We discussed XSR from logical and thermodynamic viewpoints and showed that the XOR gates in XSR increase the number of random bits by producing mutual information. Since XSR utilizes simple logic gates, it can be implemented by a variety of logic devices. As a proof of concept, we designed and demonstrated an AQFP-based XSR circuit generating four random bitstreams. The measurement results for autocorrelation and cross-correlation showed that the XSR circuit can generate multiple uncorrelated random bitstreams. Our next step is to develop energy-efficient alternative-computing systems using AQFP-based XSR circuits.

## Data Availability

All data generated or analyzed during this study are included in this article.
